# The interactome and proteomic responses of ALKBH7 in cell lines by in-depth proteomics analysis

**DOI:** 10.1186/s12953-019-0156-x

**Published:** 2019-12-29

**Authors:** Shu Meng, Shaohua Zhan, Wanchen Dou, Wei Ge

**Affiliations:** 1 State Key Laboratory of Medical Molecular Biology & Department of Immunology, Institute of Basic Medical Sciences Chinese Academy of Medical Sciences, School of Basic Medicine Peking Union Medical College, No.5 Dongdan Santiao, Dongcheng District, Beijing, 100005 China; 2 National Center for Clinical Laboratories, Beijing Hospital, National Center of Gerontology, Institute of Geriatric Medicine, Chinese Academy of Medical Sciences, Beijing, People’s Republic of China; 3 Department of Neurosurgery, Peking Union Medical College Hospital, Chinese Academy of Medical Sciences & Peking Union Medical College, Beijing, 100730 China; 4 Department of Neurosurgery, Affiliated Hospital of Hebei University, Baoding, 071000 China; 5 State Key Laboratory of Medical Molecular Biology & Department of Immunology, Institute of Basic Medical Sciences Chinese Academy of Medical Sciences, School of Basic Medicine Peking Union Medical College, Beijing, 100005 China; 6 Graduate School, Peking Union Medical College, Chinese Academy of Medical Sciences, Beijing, 100730 People’s Republic of China

**Keywords:** ALKBH7, MS/MS, Programmed necrosis, Lipid metabolism

## Abstract

**Background:**

ALKBH7 is a mitochondrial protein, involved in programmed necrosis, fatty acid metabolism, cell cycle regulation, and prostate cancer disease. However, the exact roles of ALKBH7 and the underlying molecular mechanisms remain mysterious. Thus, investigations of the interactome and proteomic responses of ALKBH7 in cell lines using proteomics strategies are urgently required.

**Methods:**

In the present study, we investigated the interactome of ALKBH7 in mitochondria through immunoprecipitation-mass spectrometry/mass spectrometry (IP-MS/MS). Additionally, we established the ALKBH7 knockdown and overexpression cell lines and further identified the differentially expressed proteins (DEPs) in these cell lines by TMT-based MS/MS. Two DEPs (UQCRH and HMGN1) were validated by western blotting analysis.

**Results:**

Through bioinformatic analysis the proteomics data, we found that ALKBH7 was involved in protein homeostasis and cellular immunity, as well as cell proliferation, lipid metabolism, and programmed necrosis by regulating the expression of PTMA, PTMS, UQCRH, HMGN1, and HMGN2. Knockdown of ALKBH7 resulted in upregulation of UQCRH and HMGN1 expression, and the opposite pattern of expression was detected in ALKBH7 overexpression cell lines; these results were consistent with our proteomics data.

**Conclusion:**

Our findings indicate that the expression of UQCRH and HMGN1 is regulated by ALKBH7, which provides potential directions for future studies of ALKBH7. Furthermore, our results also provide comprehensive insights into the molecular mechanisms and pathways associated with ALKBH7.

## Background

Genomic stability, which is threatened by a variety of endogenous and exogenous factors, is vital for the physiological activities of organisms [1]. In bacteria, the defense against alkylating reagents is executed by protein AlkB, which removes alkylating adducts by catalyzing a hydroxylation process utilizing 2-oxoglutarate and ferrous ion [2]. Nine AlkB homologs are encoded by the human genome: ALKBH1–8 and fat mass- and obesity-associated protein (FTO). The quantity suggests the existence of either functional redundancy or diversity among these eukaryotic AlkB family members [3]. Previous studies revealed that ALKBH1, ALKBH2 and ALKBH3 remove the methyl moiety from 3-methylthymine (3-meT) and/or 3-methylcytosine (3-meC) on DNA or RNA strands [4, 5]; ALKBH5 and FTO catalyze demethylation of the 6-methyladenosine (6-meA) moiety of mRNAs [6, 7]; ALKBH8 mediates the methylation and hydroxylation of tRNAs via its methyltransferase and AlkB domains, respectively [8, 9]. To date, there is no evidence that ALKBH4, ALKBH6 and alpha-ketoglutarate-dependent dioxygenase alkB homolog 7 (ALKBH7) function as nucleic acid hydroxylases [10].

The AlkB family belongs to the dioxygenase superfamily, that utilizes similar catalytic mechanisms but acts on completely different substrates [11]. It has been reported that AlkB family members act on protein substrates, such as ALKBH1 on H2A and ALKBH4 on actin K84me1 [12, 13]. But for ALKBH6 and ALKBH7, hint to their substrate type has yet to be revealed. Previous studies revealed that ALKBH7 is a mitochondrial protein and play a key role in programmed necrosis [14]. Furthermore, ALKBH7-knockout mice showed defects in lipid metabolism [15]. However, the exact roles of ALKBH7 and the underlying molecular mechanisms remain mysterious. Thus, it is urgent to globally investigate the interactome and proteomic responses of ALKBH7 in cell lines using proteomics strategies.

In the present study, we investigated the interactome of ALKBH7 in mitochondria through immunoprecipitation-mass spectrometry/mass spectrometry (IP-MS/MS). Additionally, we established the ALKBH7 knockdown and overexpression cell lines and further identified the differentially expressed proteins (DEPs) in these cell lines by Tandem mass tags (TMT)-based MS/MS.

## Materials and methods

### Cell lines and antibodies

To establish the stable ALKBH7 knockdown HeLa cells, a series of pBABE retrovirus vectors were constructed. The corresponding target sequences were listed in Additional file 1: Table S1. The retroviruses were then packaged in HEK293T cells with plasmids containing *gag* and *rev*. HeLa cells were transduced with harvested retroviruses supernatant and screened with puromycin. Knockdown efficiency was confirmed by qPCR and western blotting. Primer sequences used in qPCR are listed in Additional file 1: Table S2. The polyclonal anti-ALKBH7 was prepared by immunizing rabbits with N-terminal GST-tagged human full-length ALKBH7 in Abgent (Suzhou, China). The serum was harvested and antigen affinity-purified. Anti-FLAG (F3165) was purchased from Sigma-Aldrich. Anti-β-actin (GTX124213) was purchased from GeneTex. Anti-UQCRH (ab154803) was purchased from Abcam. Anti- HMGN1 (CSB-PA010568GA01HU) was purchased from CUSABIO.

### Immunoprecipitation (IP)

For IP lysate preparation, HeLa S3 cells were harvested and treated with hypotonic buffer. Then cytoplasm and the nuclei were separated with a tissue grinder and by centrifugation. To obtain nuclear extract, nuclei were resuspended in half the pellet volume of low salt buffer and then mixed with half a pellet volume of high salt buffer, drop by drop and with gentle swirling. After dialysis, the supernatant was collected with by centrifugation at 20,000 g ready for IP. To obtain cytosolic fraction, cytoplasm fraction was centrifuged at 17,000 g for 15 min. The supernatant was harvested and then dialyzed and centrifuged at 17,000 g for 15 min. To obtain crude mitochondrial fraction, pellet from cytoplasm fraction was lysed and centrifuged at 20,000 g for 30 min and the supernatant was harvested for further use. For IP assays, the subcellular fractions were incubated with antibody-conjugated agarose for 4 h at 4 °C. The immune-complexes were finally eluted with 0.1 M glycine, pH = 2, and then resolved in a denatured gel.

### In-gel digestion

Proteins in the crude mitochondrial fraction were captured in the IP assay and then separated by SDS-PAGE in a 4–12% gradient Noves Bis-Tris gel (Thermo Fisher Scientific, NP0321BOX). The gels were lightly stained with Coomassie brilliant blue R250 (Thermo Fisher Scientific,20,278) for 15 min. Five regions of gel with distinctive proteins bands were removed and diced into 1 mm^3^ cube, followed by in-gel digested as previously described [16]. Briefly, each gel slice was desiccated with acetonitrile, treated with 10 mM dithiothreitol (DDT) (GE Healthcare Life Sciences, 17,131,801) for 1 h at 55 °C and then with 25 mM iodoacetamide (IAA) (Amersham Biosciences, RPN6302V) for 30 min in the dark at room temperature. The gel slices were then digested overnight at 37 °C with trypsin (Promega, V5280) at a protein/protease ratio of 12.5:1 for liquid chromatography- MS/MS (LC-MS/MS).

### TMT-based quantitative proteomics

Four cell lines were used for TMT-based quantitative proteomics: transient ALKBH7 overexpressed (ALKBH7^OE^) HEK293T cells, stable ALKBH7 knockdown (shALKBH7) HeLa cells, and their corresponding control cells. Cell lines were separately harvested and treated with fresh lysis buffer (8 M urea in PBS, pH 8–8.5;1 mM PMSF; 1 mM protease inhibitor cocktail). The lysates were then reduced by incubation with 5 mM DTT at 60 °C for 1 h and alkylated by incubation with 25 mM IAA in darkness at room temperature for 30 min. Trypsin and Lys-C (Promega, V5072) were then added at a 25:1(w/w) at 37 °C for 16 h according to the manufacturers’ instructions (Thermo Fisher Scientific, 90,068). Digested peptides were acidified with 1% formic acid and desalted with a reverse-phase column (Oasis HLB, WAT094225). The extracts were dried with a vacuum concentrator and finally dissolved in 200 mM triethylammonium bicarbonate buffer for the TMT labeling. TMT isobaric label reagents (0.8 mg TMT dissolved in 40 μl 99.9% acetonitrile) were separately used to label two biological sets of the cell lines as follows: TMT-126 for shALKBH7 control Hela cell lines (Scramble), TMT-127 for shALKBH7 Hela cell lines, TMT-128 for ALKBH7^OE^ control HEK293T cell lines (Vector), TMT-129 for ALKBH7^OE^ HEK293T cell lines. All labeled peptides were then separately mixed, desalted, dried and dissolved in 100 μl 0.1% formic acid for subsequent high-performance liquid chromatography (HPLC) and LC-MS/MS analysis.

### HPLC

The TMT-labelled peptides were fractionated using an UltiMate 3000 HPLC system (Thermo Scientific) with an XBridgeTM BEH300 C18 column (Waters, 4.6 × 250 mm, 2.5 μm). Gradient elution buffers A and B were methanol and acetonitrile, respectively. Peptides were separated by gradient elution as follows: 0–8% phase B (98% acetonitrile + 2% ddH_2_0, pH 10 adjusted with NH_3_·H_2_O) for 5 min, 8–18% phase B for 35 min, 18–32% phase B for 22 min, 32–95% phase B for 6 min, and 95–0% phase B for 4 min. Fractions were collected every 90 s into 48 tubes, then dried and combined into 20 fractions. Finally, these fractions were re-dissolved in 20 μl 0.1% formic acid for LC-MS/MS analysis.

### LC-MS/MS and protein identification

For LC-MS/MS analysis, peptides were separated by a 120 min gradient elution at a flow rate 0.300 μL/min with the EASY-nLC 1000 system which was directly interfaced with the Thermo Orbitrap Fusion mass spectrometer. The analytical column was a homemade fused silica capillary column (75 μm ID, 150 mm length; Upchurch, Oak Harbor, WA) packed with C-18 resin (300 A, 5 μm; Varian, Lexington, MA). Mobile phase A consisted of 0.1% formic acid, and mobile phase B consisted of 100% acetonitrile and 0.1% formic acid. The Orbitrap Fusion mass spectrometer was operated in the data-dependent acquisition mode using Xcalibur 3.0 software. A single full-scan mass spectrum was done in the Orbitrap (350–1550 m/z, 120,000 resolution). The spray voltage is 2500 V and the automatic gain control (AGC) target is 200,000. This was followed by 3 s data-dependent MS/MS scans in an ion routing multipole at 30% normalized collision energy (HCD). The charge state screening of ions was set at 2–7. The exclusion duration was set at 15 s. Mass window for precursor ion selection was set at 1.6 m/z. The MS2 spectra were acquired with a resolution of 30,000 and maximum injection time of 60 ms.

The IP-MS/MS raw data were analysed through the Proteome Discoverer software (Version 1.4, Thermo Scientific) against the human reviewed Swiss-Prot FASTA database (released on 2016.06.19). The criteria for searching were set according to the software recommendations. Furthermore, the Proteome Discoverer software (Version 2.2, Thermo Scientific) was also used to analyse two sets of TMT-based MS/MS raw data against the human reviewed Swiss-Prot FASTA database (released on 2016.06.19). The search criteria set as follows: A maximum number of two missed trypsin/Lys-C cleavages were allowed. Precursor ion mass tolerances were set at 10 ppm for all MS acquired in an Orbitrap mass analyser and the fragment ion mass tolerance was set at 20 mmu for all MS/MS spectra acquired. Carbamidomethylation (C, + 57.021 Da) and TMT-6plex (K and peptide N-terminus) were set as fixed modifications, and oxidation (methionine, M) was specified as the variable modification. The FDR of peptide and protein identification were both set to 0.01 (1%). Relative protein quantification was based on the intensities of six reporter ion per peptide. Quantification was carried out only for proteins with two or more unique peptides matched. Protein ratios were calculated as the median of all peptide hits belonging to a protein. Finally, the IP-MS/MS and TMT-based MS/MS raw data were deposited in the ProteomeXchange Consortium via the PRIDE partner repository with the dataset identifiers PXD008318, PXD008319 and PXD008320, respectively.

### Bioinformatic analysis

The ratio of proteins (normalized against GAPDH) ≥1.3 or ≤ 0.77 were identified as DEPs. The Gene Ontology (GO) and pathway enrichment analysis of DEPs was performed using the Funrich tool (Version 3.0) [17] and visualized with JMP software (Version 14.0). Boxplot was also visualized by JMP software. The protein-protein interaction (PPI) analysis of DEPs was performed with the String website (http://string-db.org) and visualized through the Cytoscape software (Version 3.1.1). For protein correlation analysis, the identified proteins from TMT-labelled four type of cell lines were analyzed by the ‘corrplot’ and ‘psych’ package with the Pearson correlation coefficient setting in R. Furthermore, Hierarchical cluster analysis of DEPs was also carried out by the ‘iheatmapr’ package with the Complete and Euclidean method setting in R.

## Results

### Establishing of ALKBH7 knockdown Hela cell lines

To investigate the biological function of ALKBH7, we constructed several stable ALKBH7 knockdown HeLa cell lines by transfection with pBABE retrovirus shRNA vectors and prepared polyclonal anti-ALKBH7 antibody by immunization of rabbits with full-length recombinant ALKBH7. Cell lines, ‘72’ and ‘73’ were shown to exhibit over 80% knockdown efficiency (Additional file 2: Figure S1C). The anti-ALKBH7 antibody was validated with the knockdown cell lines and proven to work well in immunofluorescence (IF) and western blotting assays (Additional file 2: Figure S1A-B). Though anti-ALKBH7 antibody picked up several non-specific bands in western blotting (Additional file 2: Figure S1B), ALKBH7 was the only one enriched in IP assays with the crude mitochondrial fraction (Additional file 2: Figure S2), which demonstrated the specificity of anti-ALKBH7 antibody in IP assays. Furthermore, The IF results showed that the signal of endogenous ALKBH7 is predominantly in cytoplasm (Additional file 2: Figure S1A), which is consistent with previous studies revealing ALKBH7 localizes in mitochondria in the steady-state.

### GO and pathway enrichment analysis of proteins enriched in mitochondrion associated with ALKBH7

**Fig. 1:**
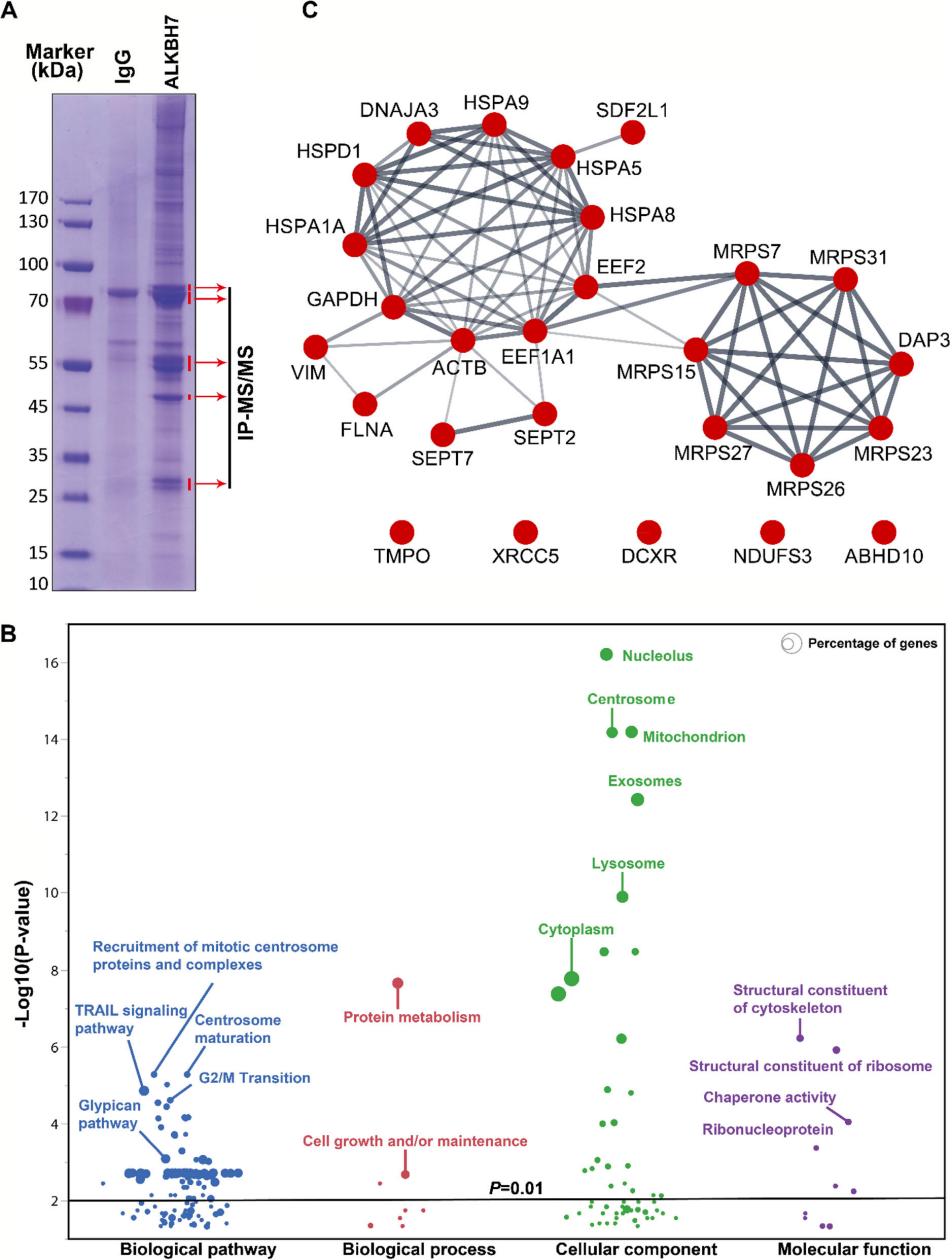
Gene Ontology and pathway enrichment analysis of proteins enriched in mitochondrion associated with ALKBH7. (**a**) ALKBH7 complexes in the crude mitochondrial extract from HeLa S3 cells were enriched with anti-ALKBH7 agarose. Five regions of gel with distinctive protein bands were excised for in-gel digestion, and further identified by MS. (**b**) Gene Ontology and pathway enrichment analysis of identified proteins detected through IP-MS/MS. The categories of biological pathway, biological process, cellular component, and molecular function are shown in blue, red, green, and purple, respectively. The percentage of genes per category is represented by the size of the node. (**c**) Protein-protein interaction network constructed with enriched proteins in mitochondrion identified through IP-MS/MS

With anti-ALKBH7 antibody, we tried to study the interactome of endogenous ALKBH7 through IP-MS/MS. The anti-ALKBH7 antibody and normal rabbit IgG were covalently conjugated with protein A agarose to prepare the anti-ALKBH7 agarose and the corresponding control, respectively. These agaroses preparations were then incubated with the crude mitochondrial lysate from HeLa S3 cells. As shown in Fig.1a, proteins captured in the IP assay were resolved in reduced denaturing electrophoresis. Five regions of gel with distinctive proteins bands were cut off and underwent in-gel digestion and MS/MS protein identification. A total of 352 proteins were identified, of which two or more unique peptides were identified for 80 proteins with a score ≥ 10. After exclusion of 11 keratins, which are accidental or unavoidable contaminants of proteomics assays, 69 proteins were converted into Entrez Gene IDs and performed for further bioinformatic analysis (Additional file 1: Table S3). Through the GO and pathway enrichment analysis, we found that 27 proteins were identified in the mitochondrion. Furthermore, the other enriched GO and pathway categories included the TRIL signaling pathway, G2/M transition, glypican pathway, protein metabolism, cell growth/maintenance, structural constituent of cytoskeleton, and chaperone activity with adjusted *p* < 0.01 by Hypergeometric test (Fig.1b, detailed information in the Additional file 1: Table S4). To further investigate the ALKBH7 binding proteins in the mitochondrion, we used the String website to analysis the PPI of the 27 proteins (Fig.1c). The majority of protein interactions converged at two hubs, including several heat shock proteins (HSPA1A, HSPD1, DNAJA3, HSPA5, HSPA8, and HSPA9) and 28S ribosomal proteins (MRPS7, MRPS15, MRPS23, MRPS26, MRPS27, MRPS31, and DAP3), which implied that ALKBH7 may be involved in the protein homeostasis. Additionally, we also considered the possibility of ALKBH7 as a proline hydrolase based on the structural similarity to PHD2 [18]. We designed 71 peptides according to the IP-MS/MS data and performed in vitro demethylation assays, however, no positive results were obtained (Additional file 1: Table S5).

### Bioinformatic analysis of DEPs in shALKBH7 and ALKBH7^OE^ cell lines through TMT-based MS/MS

**Fig. 2:**
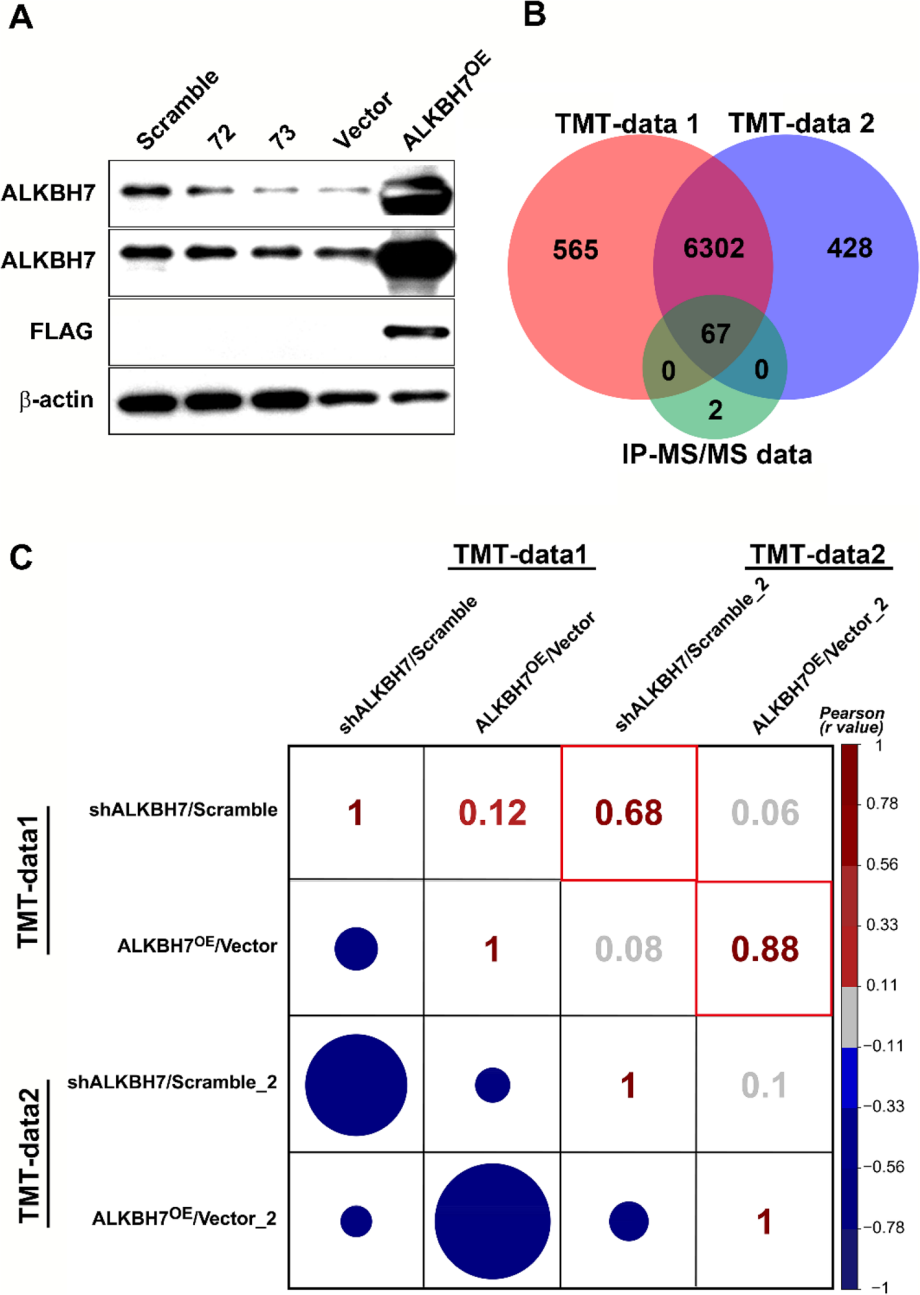
Venn diagram and correlation analysis of identified proteins in shALKBH7 and ALKBH7^OE^ cell lines through TMT-based MS/MS. (**a**) transient overexpressed ALKBH7-FLAG HEK293T cells and the stable ALKBH7 knockdown HeLa cell line ‘72’ and ‘73’. (**b**) The number of proteins unique to, or shared by, both biological replicates of TMT-based MS/MS and IP-MS/MS is shown in a Venn diagram. (**c**) Correlation matrix showing all the abundance of the identified proteins in two biological replicates of TMT-based MS/MS. Red boxes indicate correlations between biological duplicates

**Fig. 3:**
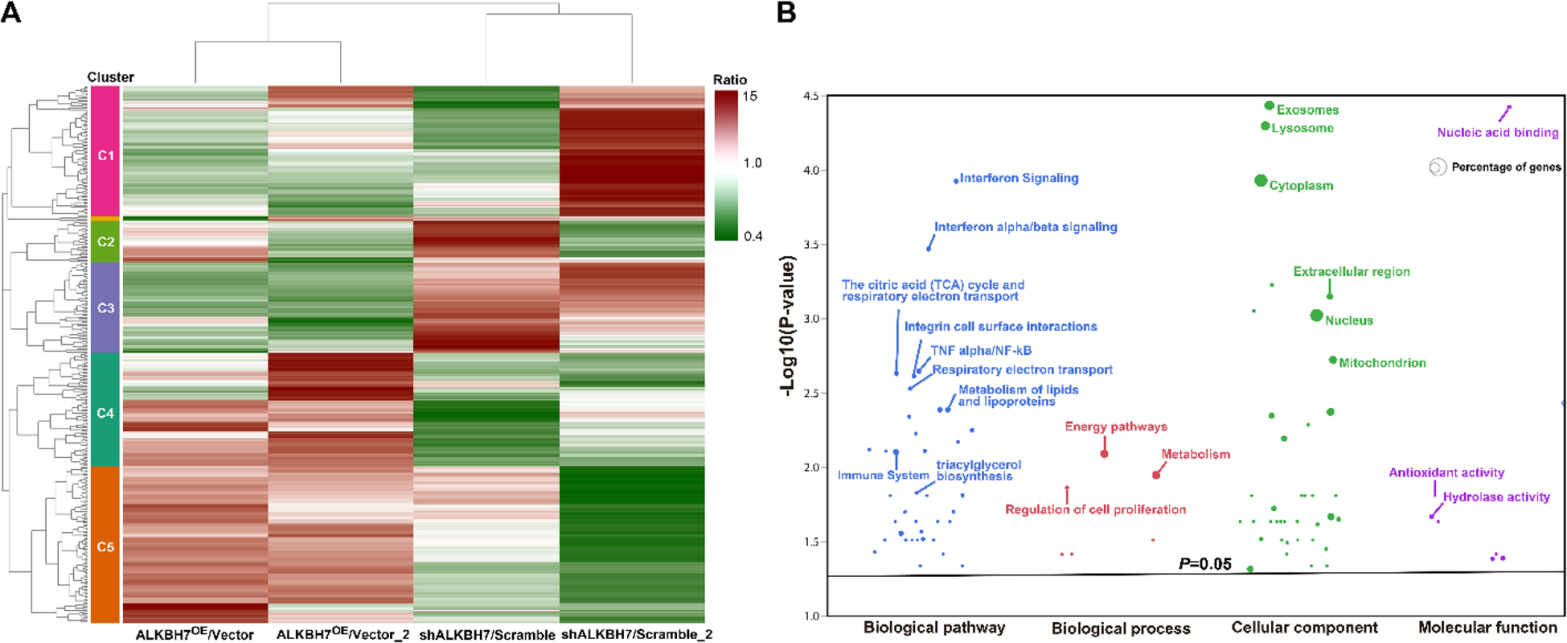
Bioinformatic analysis of differentially expressed proteins (DEPs) in shALKBH7 and ALKBH7^OE^ cell lines through TMT-based MS/MS. (**a**) Hierarchical clustering of DEPs in one or two biological replicates of TMT-based MS/MS by Heatmap. Red indicates upregulated DEPs, whereas green indicates downregulated DEPs. The panel on the left shows the five different clusters. (**b**) GO and pathway enrichment analysis of DEPs in Clusters 3 and 5. The categories of biological pathway, biological process, cellular component, and molecular function are shown in blue, red, green, and purple, respectively. The percentage of genes per category is represented by the size of the node

**Fig. 4:**
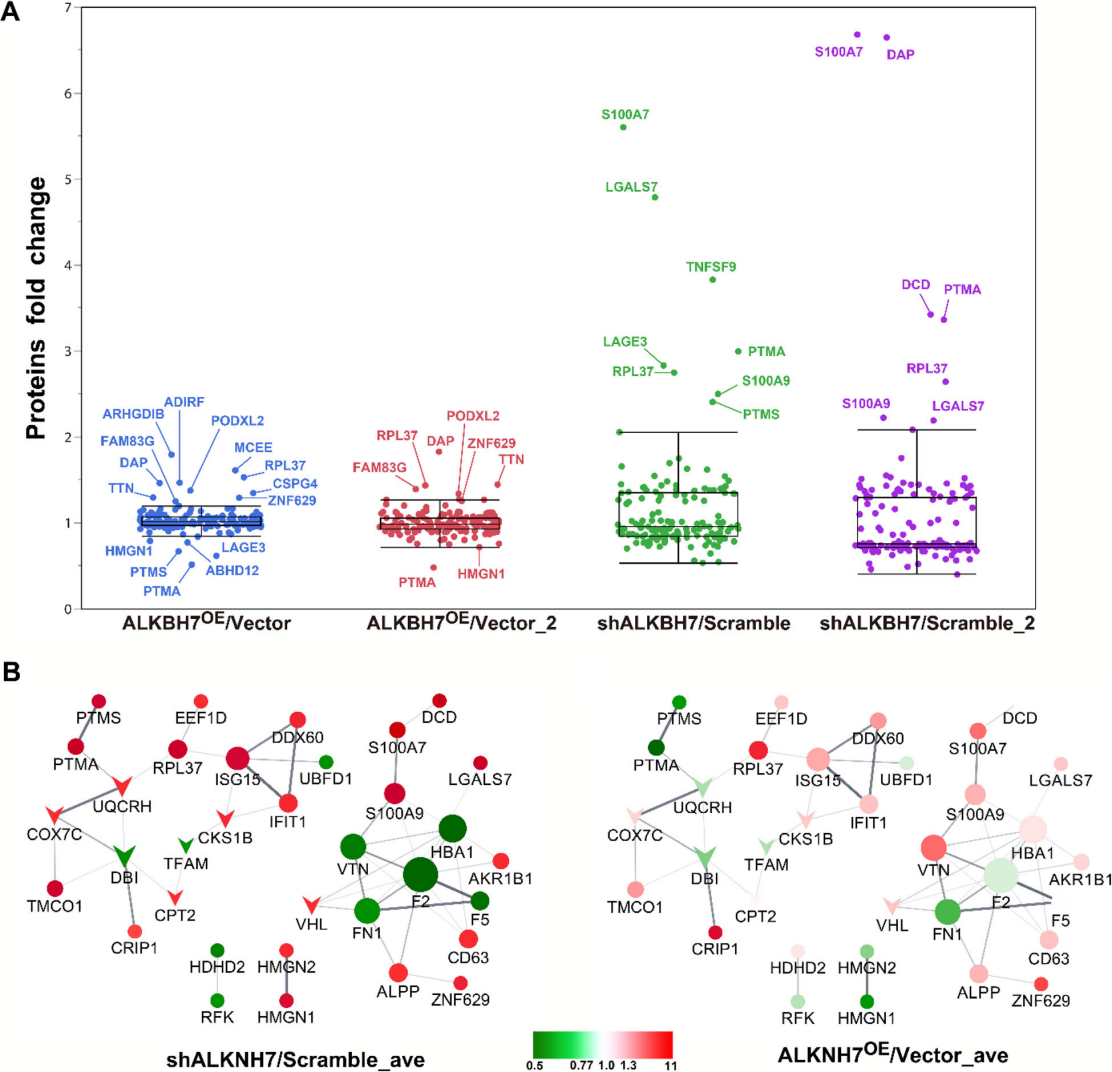
A comprehensive interaction network among the proteins changed in two biological TMT-MS/MS replicates. (**a**) Box plot denotes the fold change of DEPs in Cluster 3 and 5. Statistical outlier proteins are colored and labeled. (**b**) Protein-protein interaction network constructed with DEPs in two biological replicates of TMT-based MS/MS; no connective DEPs were excluded. DEPs are represented by colored nodes labeled with the gene name. The average fold changes of DEPs in two biological replicates of TMT-based MS/MS are reflected by color intensity. Upregulated and downregulated DEPs are shown in red and green, respectively. V-shape nodes denote DEPs located in mitochondrion. Node size correlates with the number of interactions

**Fig. 5:**
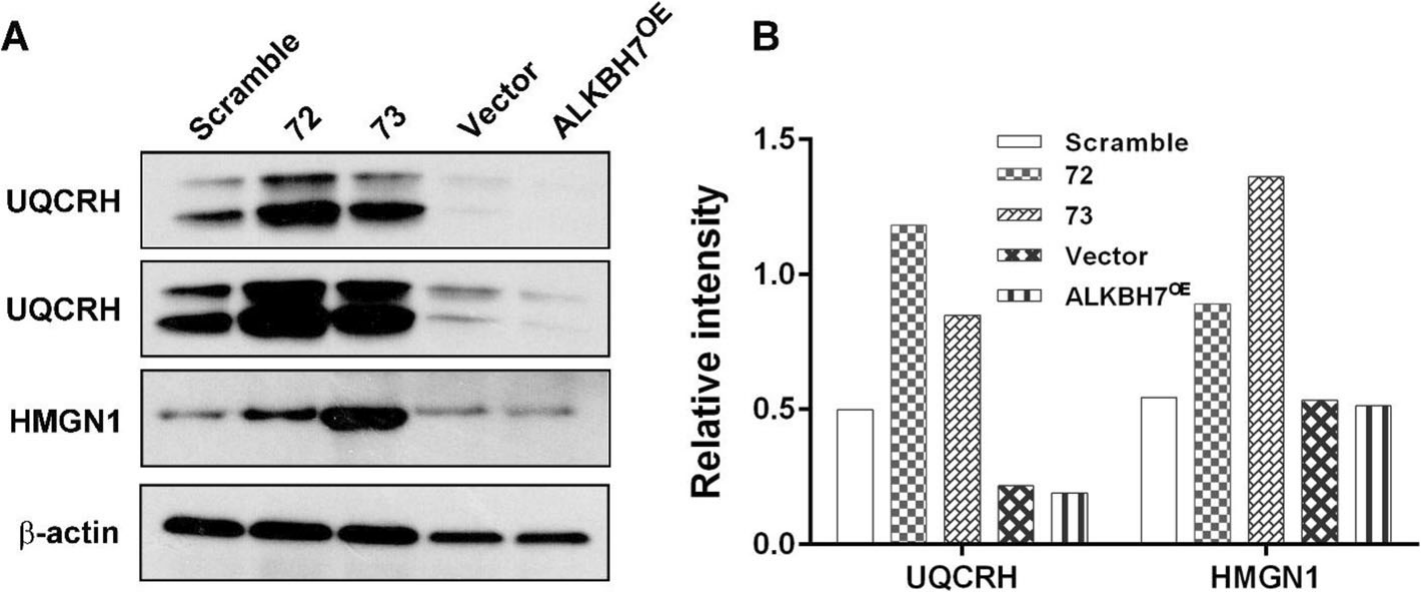
Validation of TMT-based proteomics data. (**a**) Two DEPs (UQCRH and HMGN1) identified in proteomics data were validated by western blotting. Knockdown of ALKBH7 resulted in upregulation of UQCRH and HMGN1 expression, and the opposite pattern of expression was detected in ALKBH7 overexpression cell lines, which were consistent with our proteomics data. (**b**) The relative intensity of UQCRH and HMGN1 in western blotting (normalized by corresponding β-actin expression)

We also conducted a TMT-based proteomic analysis of the effects of knockdown or overexpression of ALKBH7 (two biological replicates). We generated ALKBH7^OE^ HEK293T cells by transient overexpressed ALKBH7-FLAG and the stable ALKBH7 knockdown HeLa cell line ‘73’ (Fig.2a). Using the Proteome Discoverer software, we identified a total of 8234 and 8038 in the two biological TMT-MS/MS replicates, respectively. With the filters of strict protein confidence (q value< 0.05 and unique peptides ≥2), 6948 and 6812 proteins were selected. Notably,14 and 15 were keratins and then excluded from further analysis. Among the remaining proteins, 6369 proteins were found in both two biological TMT-MS/MS replicates and by IP-MS/MS (Fig.2b, Additional file 1: Table S6), and the ratios of these proteins were used for calculating correlations between two biological TMT-MS/MS replicates. Moderate correlation coefficients (r = 0.68) were observed between two shALKBH7/Scramble replicates, whereas high correlation coefficients (r = 0.88) were obtained between the two ALKBH7^OE^/Vector replicates (Fig.2c). These results suggested the consistency and quality of the two biological TMT-MS/MS replicates, which warranted further analysis. Through using cutoff ratios for DEPs (set as ≥1.3-fold or ≤ 0.77-fold), a total of 326 DEPs were obtained in the two biological replicates (Additional file 1: Table S6). Pairwise comparisons of these DEPs using the hierarchical clustering heatmap revealed that 50 DEPs in cluster 3 were downregulated in two ALKBH7^OE^/Vector groups and upregulated in two shALKBH7/Scramble groups, while inverse patterns of 100 DEPs were found in cluster 5 (Fig.3a, detailed information in the Additional file 1: Table S6). To gain a better insight into the biological significance of these DEPs, we also performed the GO and pathway enrichment analysis of these 150 DEPs. A total of 20 proteins, mainly participating in respiratory electron transport, were identified in the mitochondrion in the cellular component enrichment analysis (Fig.3b). Furthermore, the other enriched GO and pathway categories included the citric acid cycle and respiratory transport, metabolism of lipids and lipoproteins, triacylglycerol biosynthesis, energy pathways, regulation of cell proliferation, antioxidant activity, and hydrolase activity with adjusted *p* < 0.05 by Hypergeometric test (Additional file 1: Table S7). These results were consistent with the IP-MS/MS results. Additionally, we found that the categories of interferon signaling and immune system were also enriched, implying ALKBH7 may be involved in regulating immune function. To further clarify biologically meaningful DEPs probably involved in these categories, the gene names of all outlier DEPs were displayed in the boxplot (Fig.4a), and these outlier DEPs in cluster 3 or 5, especially PTMA, may be involved in the physiological or pathological processes of ALKBH7, albeit some proteins changing in only one replicates. Therefore, to further investigated the PPI network among the proteins changed in both two biological TMT-MS/MS replicates, a comprehensive interaction network of the 55 DEPs was performed through the String website and Cytoscape software (Fig.4b, detail information in the Additional file 1: Table S8). We found that the following DEPs have different changing patterns in the shALKBH7 and ALKBH7^OE^ cell lines, including PTMA, PTMS, UQCRH, HMGN1 and HMGN2, especially UQCRH located in the mitochondrion. To further validate our proteomics data, we performed western blotting analysis of UQCRH and HMGN1 in ALKBH7 knockdown and overexpression cell lines. Knockdown of ALKBH7 overexpressed the UQCRH and HMGN1 expression, and vice versa in ALKBH7 overexpression cell lines (Fig.5a-b), The patterns of UQCRH and HMGN1 expression in the western blotting were concordant with our proteomics data, which further validate our bioinformatic results.

## Discussion

We performed IP-MS/MS and TMT-based MS/MS analyses to further investigate the physiological or pathological processes of ALKBH7 in mammals. GO and pathway enrichment analyses revealed that ALKBH7 is associated with protein metabolism, cell growth and/or maintenance, the citric acid cycle and respiratory electron transport, triacylglycerol biosynthesis, antioxidant activity, and structural constituent of the cytoskeleton. These results were consistent with the previous reports that ALKBH7 is associated with programmed necrosis, fatty acid metabolism, cell cycle regulation, and prostate cancer disease [14, 15, 19, 20] and suggest the reliability of our proteomics data. Furthermore,13 identified interacting proteins of ALKBH7 in the mitochondrion by IP-MS/MS were heat shock proteins and 28S ribosomal proteins, suggesting that ALKBH7 may be involved in the protein homeostasis. Notably, biology pathway analysis revealed that interferon signaling and immune system were enriched with DEPs upon different ALKBH7 expression in cell lines by TMT-based MS/MS, implying ALKBH7 may also be involved in cellular immunity. However, these results need to be in-depth investigated in the future.

In TMT-based MS/MS, we also found that the expression of PTMA, PTMS, UQCRH, HMGN1, and HMGN2 proteins were upregulated in shALKBH7 cell lines whereas downregulated in ALKBH7^OE^ cell lines. PTMA and PTMS have similar sequences, comprising 101 and 111 amino acid residues, respectively [21, 22]. Previous study has demonstrated that PTMA is associated with cell division and proliferation, immunomodulatory activity, and acetylation of histones [23–25], while PTMS was involved in modulating cellular immunity, binding to the linker histone H1 and remodeling chromatin structure [26–28]. UQCRH, which is located in the mitochondria, is required for the electron transfer between cytochrome c and cytochrome c1 during oxidative phosphorylation [29]. It has also been demonstrated that UQCRH is important for the intracellular production of reactive oxygen species (ROS) [30, 31]. These results were reminiscent of the previous study that ALKBH7 is required for programmed necrosis through inhibiting mitochondrial function, such as the formation of ROS [14]. Interesting, HMGN1 and HMGN2 are a family of nuclear proteins that bind specially to nucleosomes and modulate chromatin structure [32]. Several review articles summarized that HMGN proteins also affect cellular differentiation and development, as well as the ability to promote a variety of DNA-related processes, such as transcription, replication, recombination and DNA damage due to both ultraviolet light and ionizing irradiation [32–34]. All these results indicate that ALKBH7 may participate in cell proliferation, metabolism of lipids and lipoproteins, and programmed necrosis possibly by directly or indirectly influencing the expression of the five DEPs.

## Conclusion

In summary, to the best our knowledge, this is the first study to globally investigate the interactome and proteomic responses of ALKBH7 in cell lines using proteomics strategies. IP-MS/MS and TMT-based MS/MS analyses indicated that ALKBH7 may be involved in both the protein homeostasis and cellular immunity, as well as cell proliferation, lipid metabolism, and programmed necrosis through regulating the expression of PTMA, PTMS, UQCRH, HMGN1, and HMGN2 proteins. Additionally, the expression of UQCRH and HMGN1 was validated in ALKBH7 knockdown and overexpression cell lines by western blotting. All these results provide comprehensive insights into the molecular mechanisms and pathways associated with ALKBH7, as well as highlighting potential directions for future research into ALKBH7.

## Supplementary information

**Additional file 1: Table S1.** Target sequences of ALKBH7. **Table S2.** Primer sequences of ALKBH7 and GAPDH used in qPCR. **Table S3.** Identified proteins in IP assay for bioinformatic analysis. **Table S4.** Enriched Gene ontology and pathway categories in IP assay. **Table S5.** Sequences of 71 peptides performed in vitro demethylation assays. **Table S6.** Identified proteins and corresponding bioinformatic analysis in TMT-based MS/MS. **Table S7.** Enriched Gene ontology and pathway categories in TMT-based MS/MS. **Table S8.** Detailed information of proteins for protein-protein interaction.

**Additional file 2: Figure S1.** ALKBH7 knockdown cell lines and custom anti-ALKBH7. **Figure S2.** anti-ALKBH7 enriched only ALKBH7 in the crude mitochondrial lysate of HeLa S3 cells

## Data Availability

All data generated or analyzed during this study are included in this published article [and its Additional files].

## Abbreviations

ALKBH7: Alpha-ketoglutarate-dependent dioxygenase alkB homolog 7, mitochondrial
DDT: Dithiothreitol
DEPs: Differentially expressed proteins
GO: Gene ontology
HMGN1: Non-histone chromosomal protein HMG-14
HMGN2: Non-histone chromosomal protein HMG-17
HPLC: High-performance liquid chromatography
IAA: Iodoacetamide
IP: Immunoprecipitation
LC-MS/MS: Liquid chromatography-tandem mass spectrometry
PPI: Protein-protein interaction
PTMA: Prothymosin alpha
PTMS: Parathymosin
TMT: Tandem mass tags
UQCRH: Cytochrome b-c1 complex subunit 6, mitochondrial
