# Supplementary material for: The interactome and proteomic responses of ALKBH7 in cell lines by in-depth proteomics analysis
Source: Proteome Sci. 2019 Dec 29;17:8. doi: 10.1186/s12953-019-0156-x (PMC6935500; doi:10.1186/s12953-019-0156-x)
Supplement: Supplementary file 2 — Additional file 2: Figure S1. ALKBH7 knockdown cell lines and custom anti-ALKBH7. Figure S2. anti-ALKBH7 enriched only ALKBH7 in the crude mitochondrial lysate of HeLa S3 cells [file 12953_2019_156_MOESM2_ESM.docx]

**Supporting information**

**The interactome and proteomic responses of ALKBH7 in cell lines by in-depth proteomics analysis**

Shu Meng^1*^, Shaohua Zhan^1, 2*^, Wanchen Dou^3#^, Wei Ge^1, 4#^

1 State Key Laboratory of Medical Molecular Biology & Department of Immunology, Institute of Basic Medical Sciences Chinese Academy of Medical Sciences, School of Basic Medicine Peking Union Medical College, No.5 Dongdan Santiao, Dongcheng District, Beijing, 100005, China.

2 National Center for Clinical Laboratories, Beijing Hospital, National Center of Gerontology, Beijing 100730, China.

3 Department of Neurosurgery, Peking Union Medical College Hospital, Chinese Academy of Medical Sciences & Peking Union Medical College, Beijing, China.

4 Department of Neurosurgery, Affiliated Hospital of Hebei University, Baoding 071000, China.

^*^ These authors contributed equally to this work.

**Supporting Information**

**Additional file 2: Figure.S1.** ALKBH7 knockdown cell lines and custom anti-ALKBH7. **Figure.S2.** anti-ALKBH7 enriched only ALKBH7 in the crude mitochondrial lysate of HeLa S3 cells.


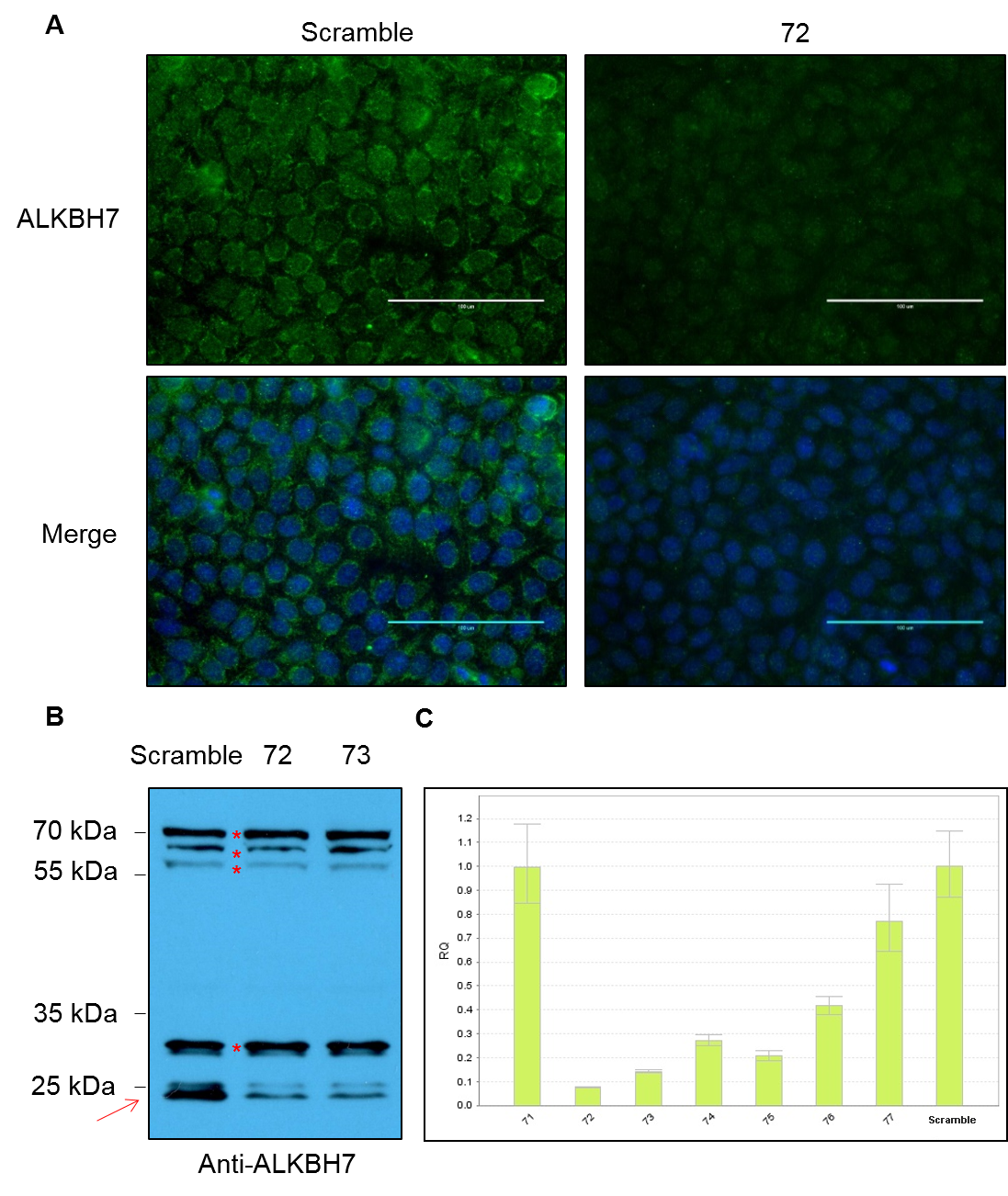


**Figure.S1.** ALKBH7 knockdown cell lines and custom anti-ALKBH7. (A) Confirmation that anti-ALKBH7 detects endogenous ALKBH7 in the cytoplasm by immunofluorescence. (B) Western blot validation of anti-ALKBH7 showing detection of endogenous ALKBH7 (red arrow). Asterisks mark the non-specific bands. (C) Normalized expression of ALKBH7 relative to GAPDH of several ALKBH7 shRNA stable cell lines analyzed by real-time qPCR. Cell lines 72 and 73 showed over 80% knockdown efficiency. 71–77: stable cell lines bearing different target sequences.


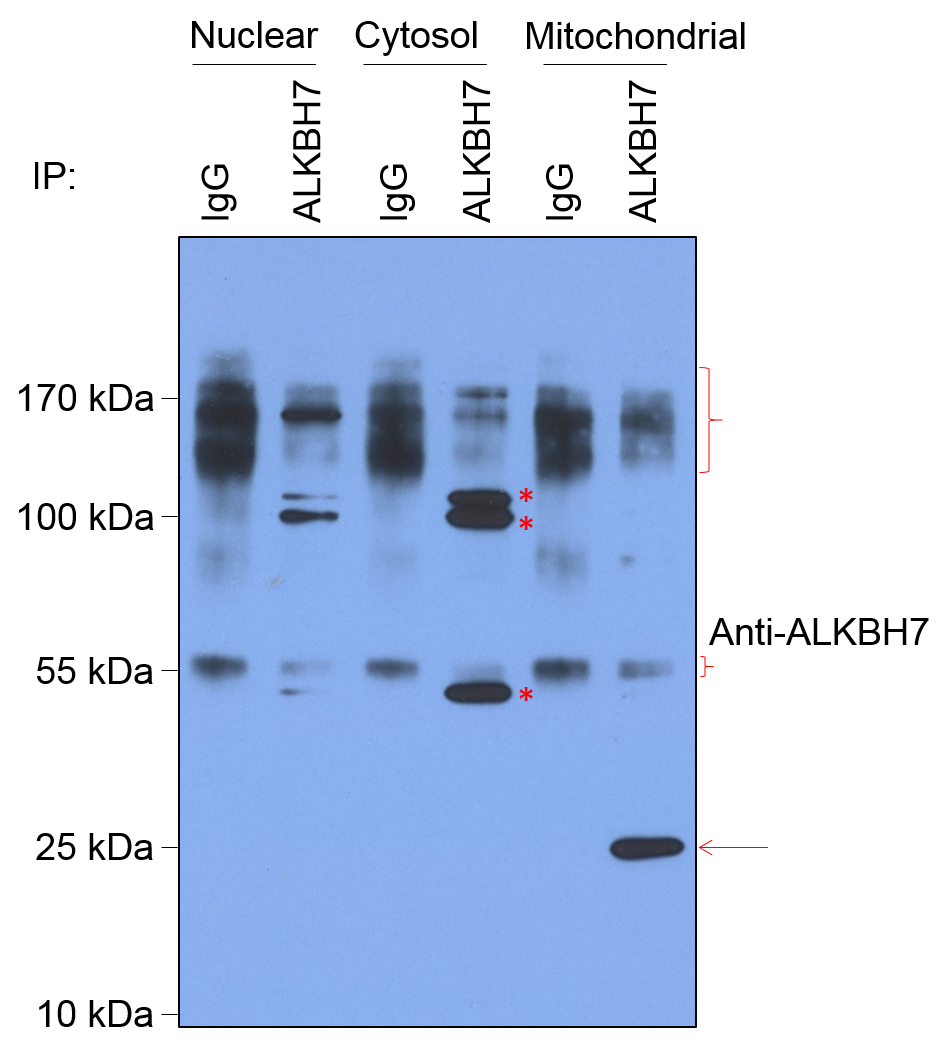


**Figure.S2**. Anti-ALKBH7 enriched only ALKBH7 in the crude mitochondrial lysate of HeLa S3 cells. Asterisks mark the non-specific capture in nuclear and cytosolic fractions. Braces mark the signals of immunoprecipitation antibodies.
